# Association between Inflammation and Cardiac Geometry in Chronic Kidney Disease: Findings from the CRIC Study

**DOI:** 10.1371/journal.pone.0124772

**Published:** 2015-04-24

**Authors:** Jayanta Gupta, Elizabeth A. Dominic, Jeffrey C. Fink, Akinlolu O. Ojo, Ian R. Barrows, Muredach P. Reilly, Raymond R. Townsend, Marshall M. Joffe, Sylvia E. Rosas, Melanie Wolman, Samir S. Patel, Martin G. Keane, Harold I. Feldman, John W. Kusek, Dominic S. Raj, the CRIC Study Investigators

**Affiliations:** 1 Department of Biomedical Sciences, Texas Tech University Health Sciences Center, El Paso, Texas, United States of America; 2 The George Washington University School of Medicine, Washington, DC, United States of America; 3 Division of Nephrology, University of Maryland School of Medicine, Baltimore, Maryland, United States of America; 4 Division of Nephrology, University of Michigan, Ann Arbor, Michigan, United States of America; 5 Cardiovascular Institute, University of Pennsylvania, Philadelphia, Pennsylvania, United States of America; 6 Renal and Electrolyte Division, University of Pennsylvania, Philadelphia, Pennsylvania, United States of America; 7 Center for Clinical Epidemiology and Biostatistics, University of Pennsylvania, Philadelphia, Pennsylvania, United States of America; 8 Joslyn Diabetic Center, Harvard Medical School, Boston, Massachusetts, United States of America; 9 Division of Renal Diseases and Hypertension, The George Washington University, Washington, DC, United States of America; 10 Department of Medicine, Temple University, Philadelphia, Pennsylvania, United States of America; 11 Division of Kidney, Urologic, and Hematologic Diseases, The National Institute of Diabetes and Digestive and Kidney Diseases, Bethesda, Maryland, United States of America; University of Florida, UNITED STATES

## Abstract

**Background:**

Left ventricular hypertrophy (LVH) and myocardial contractile dysfunction are independent predictors of mortality in patients with chronic kidney disease (CKD). The association between inflammatory biomarkers and cardiac geometry has not yet been studied in a large cohort of CKD patients with a wide range of kidney function.

**Methods:**

Plasma levels of interleukin (IL)-1β, IL-1 receptor antagonist (IL-1RA), IL-6, tumor necrosis factor (TNF)-α, transforming growth factor (TGF)-β, high-sensitivity C-Reactive protein (hs-CRP), fibrinogen and serum albumin were measured in 3,939 Chronic Renal Insufficiency Cohort study participants. Echocardiography was performed according to the recommendations of the American Society of Echocardiography and interpreted at a centralized core laboratory.

**Results:**

LVH, systolic dysfunction and diastolic dysfunction were present in 52.3%, 11.8% and 76.3% of the study subjects, respectively. In logistic regression analysis adjusted for age, sex, race/ethnicity, diabetic status, current smoking status, systolic blood pressure, urinary albumin- creatinine ratio and estimated glomerular filtration rate, hs-CRP (OR 1.26 [95% CI 1.16, 1.37], p<0.001), IL-1RA (1.23 [1.13, 1.34], p<0.0001), IL-6 (1.25 [1.14, 1.36], p<0.001) and TNF-α (1.14 [1.04, 1.25], p = 0.004) were associated with LVH. The odds for systolic dysfunction were greater for subjects with elevated levels of hs-CRP (1.32 [1.18, 1.48], p<0.001) and IL-6 (1.34 [1.21, 1.49], p<0.001). Only hs-CRP was associated with diastolic dysfunction (1.14 [1.04, 1.26], p = 0.005).

**Conclusion:**

In patients with CKD, elevated plasma levels of hs-CRP and IL-6 are associated with LVH and systolic dysfunction.

## Introduction

Left ventricular hypertrophy (LVH) increases the risk of cardiovascular (CV) mortality and morbidity in the general population as well as in patients with chronic kidney disease (CKD).[1,2] Although LVH begins as an adaptive response to pressure or volume overload, it often results in diastolic dysfunction, eventually leading to heart failure. Abnormal cardiac geometry in patients with CKD has been attributed to a number of established risk factors as well as risk factors unique to CKD.[3,4] Evidence from experimental studies indicates that cytokines regulate cardiac remodeling and contractile function.[5,6] However, to date, no large scale study has examined the association between biomarkers of inflammation and cardiac geometry in a multiracial cohort of subjects with established CKD. Understanding the role of inflammatory molecules in the pathogenesis of heart disease in CKD is important for the design and implementation of targeted anti-inflammatory therapies.

We recently reported that biomarkers of inflammation were inversely associated with measures of kidney function and positively with the magnitude of proteinuria in chronic renal insufficiency cohort (CRIC) study participants.[7] In the same cohort, Park et al [8] found that the risk of LVH was increased among subjects with a cystatin based estimated glomerular filtration rate (eGFR) of less than 30 ml/min per 1.73 m^2^. In the present study, we examined whether systemic inflammation is a predictor of cardiac structure and function independent of the level of kidney function.

## Materials and Methods

The CRIC study is an ongoing, multicenter, prospective observational cohort study of men and women with CKD. The design of the CRIC study has been previously reported.[9] All of the 3,939 study participants have provided written informed consent. The study complies with the Declaration of Helsinki and the protocol was approved by the Institutional Review Board at each participating site (University of Pennsylvania, Philadelphia, PA; Johns Hopkins Medicine, Baltimore, MD; University of Maryland, College Park, MD; University Hospitals Case Medical Center, Cleveland, OH; MetroHealth System, Cleveland, OH; Cleveland Clinic Foundation, Cleveland, OH; University of Michigan, Ann Arbor, MI; St. John Hospital and Medical Center, Grosse Pointe Woods, MI; Wayne State University, Detroit, MI; University of Illinois at Chicago, Chicago, IL; Tulane University, New Orleans, LA; Kaiser Foundation Research Institute, Oakland, CA).

### CRIC data collection

Demographic characteristics, medical history, smoking status, weight, height, body mass index (BMI) and use of medications including statins, angiotensin converting enzyme inhibitors (ACEI) and angiotensin receptor blockers (ARB) were recorded at baseline. Serum creatinine was measured by the Jaffe method on a Beckman Synchron System. eGFR was computed using the Modification of Diet in Renal Disease estimating equation.[10] Proteinuria was measured as the ratio of albumin to creatinine in the urine (UACR).

### Measurement of biomarkers of inflammation

Biomarker measurements were performed as described earlier.[7] Briefly, high sensitivity sandwich ELISAs (Quantikine HS, R&D Systems, Minneapolis, MN) were used to measure plasma interleukin (IL)-1β, IL-6, and tumor necrosis factor (TNF)-α levels. Standard sandwich ELISAs (Quantikine, R&D Systems) were used to quantify IL-1 receptor antagonist (IL-1RA) and transforming growth factor (TGF)-β levels. Integrated performance of IL-1β, IL-1RA, IL-6, and TNF-α ELISAs were implemented using a robotic liquid handling platform (Biomek FXp, Beckman Coulter, Brea, CA). All cytokine assays were performed in duplicates and mean values used in the analysis. High sensitivity C-reactive protein (hs-CRP) and fibrinogen were quantified in EDTA plasma samples using specific laser-based immunonephelometric methods on the BNII (Siemens Healthcare Diagnostics, Deerfield, IL).

### Echocardiography

Echocardiography was performed on all study participants within 14 months of enrollment in the study. Studies were performed according to the recommendations of the American Society of Echocardiography and interpreted at a centralized, quality-controlled quantitative echocardiography core laboratory.[11] LV mass was calculated using the area–length method and indexed to height^2.7^ (LVMI).[11,12] LVH was defined as LV mass/height^2.7^ ≥ 47 g/m^2.7^ in women and ≥50 g/m^2.7^ in men.[13] Relative wall thickness (RWT) was calculated as 2 × posterior wall thickness/LV internal linear dimension in diastole. Based on the LVMI and RWT measurements,[11] four geometric patterns were described:

- normal (normal LVMI and normal RWT)
- concentric remodeling (normal LVMI and increased RWT)
- eccentric hypertrophy (abnormally increased LVMI and normal RWT), and
- concentric hypertrophy (abnormally increased LVMI and increased RWT).

Mitral inflow E- and A-wave velocities, E-wave deceleration time, and pulmonary venous reverse A-wave duration were used to categorize LV diastolic function, using well-established criteria.[14] LV end-diastolic volume (LVEDV) and LV end-systolic volume (LVESV) were calculated using the modified biplane method. Ejection fraction was calculated as (LVEDV-LVESV)/LVEDV. Systolic dysfunction was defined as ejection fraction <45%.[8,15]

### Statistical Analyses

Descriptive statistics for selected demographic and clinical characteristics of the study population stratified by the presence of LVH are presented. Values are presented as frequency (percentage), mean (standard deviation; SD) and median (inter-quartile range; IQR) as appropriate. Two sample t-test, Wilcoxon rank-sum test and Pearson’s chi-squared test were used to compare continuous and categorical variables across the LVH strata. The association of inflammatory biomarkers with the presence of LVH, systolic dysfunction and diastolic dysfunction were examined with logistic regression models. Multinomial logistic regression was used to examine the association between inflammatory biomarkers and cardiac geometry (the outcomes of concentric hypertrophy, concentric remodeling, and eccentric hypertrophy, with normal cardiac geometry as reference). Linear regression models were used to investigate the association of the inflammatory biomarkers with LVMI and ejection fraction. All models were adjusted for age, sex, race/ethnicity, diabetic status, current smoking status, systolic blood pressure, eGFR and log transformed UACR. The addition of ACEI-ARB use as a covariate did not influence the effect estimates and p-values and therefore it was not included in the final models. All principal predictors (hs-CRP, fibrinogen, serum albumin, IL-1β, IL-1RA, IL-6, TNF-α and TGF-β) were log-transformed and expressed in standard deviation units for use in regression analyses. Bonferroni’s correction was used to adjust for multiple comparisons. All analyses were performed using the SAS statistical software (version 9.3; SAS Inc., Cary, NC).

## Results

Demographic and clinical characteristics of the participants categorized by the presence of LVH are shown in **Table 1.** LVH was present in 1,631 (52.3%) participants and 16.8% of those with LVH had ejection fraction <45%. Those with LVH were more likely to be female, non-Hispanic Black, older in age, former smoker, diabetic, hypertensive and with reduced kidney function when compared to those without LVH. As a group, they also had a higher systolic BP and a larger BMI, and were more likely to report ACEI-ARB use than the group without LVH. Subjects with LVH also had lower levels of serum albumin and higher levels of inflammatory biomarkers (except TGF-β) on an average compared to those without LVH.

**Table 1:** Baseline demographic and clinical characteristics of the study cohort categorized by the presence of LVH.

Variables presented as n (%), mean ± standard deviation and median (interquartile range) as appropriate	LVH absent n = 1488	LVH present n = 1631	p-value
Females	647 (43.5)	785 (48.1)	0.009
Race/ethnicity			<0.001
Non-Hispanic white	796 (53.5)	499 (30.6)	
Non-Hispanic Black	512 (34.4)	790 (48.4)	
Hispanic	117 (7.9)	281 (17.2)	
Other	63 (4.2)	61 (3.7)	
Diabetes	524 (35.2)	956 (58.6)	<0.001
Hypertension	1153 (77.5)	1522 (93.3)	<0.001
Ever smoked	753 (50.6)	907 (55.6)	0.005
Smoke now	174 (11.7)	206 (12.6)	0.42
ACEI-ARB use	957 (64.6)	1169 (72.2)	<0.001
Age (years)	55.7 ± 11.6	59 ± 10.2	<0.001
Systolic BP (mmHg)	121.7 ± 18.8	134 ± 22.5	<0.001
Diastolic BP (mmHg)	71.2 ± 11.8	72.1 ± 13.5	0.07
BMI (kg/m^2^)	28.8 ± 5.8	34 ± 7.5	<0.001
eGFR (ml/min/1.73m2	46.1±13.5	40.2 (13)	<0.001
UACR (mcg/mg)	21.1 (5.9, 203.8)	109.2 (14.7, 868.2)	<0.001
Total cholesterol (mmol/L)	4.8 ± 1.11	4.7± 1.2	0.09
Hemoglobin (g/L	130 ± 17.0	122 ±18.0	<0.001
hs-CRP (nmol/L)	18.1 (8.6, 44.8)	27.6 (11.4, 66.7)	<0.001
Fibrinogen (μmol/L)	11.2 (9.4, 12.9)	12.6 (10.6, 14.7)	<0.001
Albumin (g/L)	41.0 (38.1, 43.0)	39.0 (36.0, 42.0)	<0.001
IL-1β (pg/ml)	0 (0, 0.9)	0.3 (0, 1.5)	<0.001
IL-1RA (pg/ml)	599.7 (328.0, 1298.5)	809.2 (424.7, 1646.7)	<0.001
IL-6 (pg/ml)	1.4 (0.9, 2.4)	2.1 (1.4, 3.3)	<0.001
TNF-α (pg/ml)	1.9 (1.3, 2.8)	2.4 (1.7, 3.4)	<0.001
TGF-β (pg/ml)	10.7 (6.2, 18.1)	10.8 (6.5, 17.6)	0.7

LVH was defined as LV mass/height^2.7^≥ 47 g/m^2.7^ in women and ≥50 g/m^2.7^ in men.UACR = Urine albumin to creatinine ratio

In multivariable linear regression analyses, hs-CRP [regression coefficient = 1.50 (95% Confidence intervals: 1.05, 1.96), p<0.001], IL-1β [0.76 (0.30, 1.23), p = 0.0010], IL-1RA [1.30 (0.84, 1.75), p<0.0001], IL-6 [1.53 (1.05, 2.01), p<0.001] and TNF-α [0.72 (0.23, 1.21), p = 0.004] were associated with LVMI (**Table 2**). Serum albumin level was negatively associated with LVMI [-1.24 (-1.76,-0.71), p<0.001]. hs-CRP [-0.75 (-1.05, -0.46), p<0.001] and IL-6 [-0.91 (-1.22, -0.60), p<0.001] were both negatively associated with ejection fraction.

**Table 2:** Adjusted linear regression models showing the association of inflammatory biomarkers with LVMI and ejection fraction.

Predictor	Outcome			
	LVMI; n = 3,119		Ejection Fraction %; n = 3,484	
	Regression coefficient (95% CI)	p-value	Regression coefficient (95% CI)	p-value
hs-CRP	1.5 (1.05, 1.96)	<0.001*	-0.75 (-1.05, -0.46)	<0.001*
Fibrinogen	0.2 (-0.27, 0.67)	0.41	-0.2 (-0.51, 0.10)	0.19
Albumin	-1.24 (-1.76,-0.71)	<0.001*	0.03 (-0.31, 0.37)	0.84
IL-1β	0.76 (0.30, 1.23)	0.001*	-0.25 (-0.55, 0.05)	0.11
IL-1RA	1.3 (0.84, 1.75)	<0.001*	-0.18 (-0.47, 0.12)	0.24
IL-6	1.53 (1.05, 2.01)	<0.001*	-0.91 (-1.22, -0.60)	<0.001*
TNF-α	0.72 (0.23, 1.21)	0.004*	-0.12 (-0.44, 0.19)	0.44
TGF-β	-0.38 (-0.83, 0.07)	0.1	0.04 (-0.25, 0.33)	0.77

Left ventricular mass index (LVMI) was calculated using the area–length method and indexed to height^2.7^

* Significant after Bonferroni correction for multiple comparisons (corrected p-value: 0.05/8 = 0.006).

LVH, systolic dysfunction and diastolic dysfunction were present in 1,631 (52.3%), 411 (11.8%) and 2,330 (76.3%) of the participants, respectively. (**Table 3**) Adjusted logistic regression analysis showed that hs-CRP [Odds Ratio = 1.26 (95% Confidence intervals: 1.16, 1.37), p<0.001), IL-1RA [1.23 (1.13, 1.34), p<0.001], IL-6 [1.25 (1.14, 1.36), p<0.001], and TNF-α [1.14 (1.04, 1.25), p = 0.004] were associated with the presence of LVH. The odds for having systolic dysfunction were greater for higher levels of hs-CRP [1.32 (1.18, 1.48), p<0.001] and IL-6 [1.34 (1.21, 1.49), p<0.001]. Only hs-CRP [1.14 (1.04, 1.26), p = 0.005] was associated with the presence of diastolic dysfunction.

**Table 3:** Adjusted logistic regression models showing the association of inflammatory biomarkers with LVH, systolic dysfunction and diastolic dysfunction.

Predictor	Outcome					
	Left ventricular hypertrophy; n = 3,119		Systolic dysfunction; n = 3,484		Diastolic dysfunction; n = 3,053	
	Odds ratio (95% CI)	p-value	Odds ratio (95% CI)	p-value	Odds ratio (95% CI)	p-value
hs-CRP	1.26 (1.16, 1.37)	<0.001*	1.32 (1.18, 1.48)	<0.001*	1.14 (1.04, 1.26)	0.005*
Fibrinogen	1.07 (0.98, 1.17)	0.14	1.02 (0.91, 1.15)	0.76	1.12 (1.02, 1.23)	0.01
Albumin	0.88 (0.80, 0.97)	0.009	0.97 (0.86 1.09)	0.57	1 (0.9, 1.11)	0.94
IL-1β	1.11 (1.03 1.21)	0.01	1.04 (0.93, 1.17)	0.47	1.04 (0.95, 1.15)	0.39
IL-1RA	1.23 (1.13, 1.34)	<0.001*	1.04 (0.94 1.16)	0.45	1.1 (1, 1.21)	0.05
IL-6	1.25 (1.14, 1.36)	<0.001*	1.34 (1.21, 1.49)	<0.001*	1.06 (0.95, 1.17)	0.3
TNF-α	1.14 (1.04, 1.25)	0.004*	1.09 (0.97, 1.23)	0.14	1.12 (1.01, 1.24)	0.03
TGF-β	0.92 (0.85, 1.00)	0.05	1.07 (0.96, 1.19)	0.24	1.01 (0.92, 1.11)	0.78

LVH was defined as LV mass/height^2.7^≥ 47 g/m^2.7^ in women and ≥50 g/m^2.7^ in men

Systolic dysfunction was defined as ejection fraction <45%

* Significant after Bonferroni correction for multiple comparisons (corrected p-value: 0.05/8 = 0.006).

Concentric remodeling, concentric hypertrophy and eccentric hypertrophy were present in 855 (28.6%), 1,102 (36.9%) and 447 (15.0%) participants, respectively. (**Table 4**) When inflammatory markers were examined for their associations with cardiac geometry using an adjusted multinomial logistic regression model, hs-CRP [Odds Ratio = 1.32 (95% Confidence Intervals: 1.17, 1.49), p<0.001], IL-1β [1.24 (1.1, 1.4), p<0.001], IL-1RA [1.4 (1.24, 1.58), p<0.001] and IL-6 [1.29 (1.13, 1.47), p<0.001] were each associated with concentric hypertrophy. Only TGF-β was associated with a higher risk of concentric remodeling [1.21 (1.09, 1.35), p<0.001]. Eccentric hypertrophy was positively associated with hs-CRP ([1.38 (1.20, 1.58), p<0.001], IL-1RA [1.24 (1.08, 1.43), p = 0.002] and IL-6 [1.31 (1.13, 1.52), p<0.001], but negatively associated with serum albumin level [0.78 (0.67, 0.91), p = 0.002].

**Table 4:** Adjusted multinomial logistic regression models showing the association of inflammatory biomarkers with cardiac geometry.

Predictor	Outcome					
	Concentric hypertrophy; n = 1,102		Concentric remodeling; n = 855		Eccentric hypertrophy; n = 447	
	Odds ratio (95% CI)	p-value	Odds ratio (95% CI)	p-value	Odds ratio (95% CI)	p-value
hs-CRP	1.32 (1.17, 1.49)	<0.001*	1.10 (0.98, 1.24)	0.1	1.38 (1.20, 1.58)	<0.001*
Fibrinogen	1.02 (0.91, 1.15)	0.76	1.00 (0.90, 1.12)	0.95	1.2 (1.01, 1.43)	0.03
Albumin	0.93 (0.81, 1.07)	0.29	0.99 (0.86, 1.13)	0.85	0.78 (0.67, 0.91)	0.002*
IL-1β	1.24 (1.1, 1.4)	<0.001*	1.08 (0.97, 1.22)	0.17	1.09 (0.95, 1.25)	0.23
IL-1RA	1.40 (1.24, 1.58)	<0.001*	1.15 (1.02, 1.29)	0.02	1.24 (1.08, 1.43)	0.002*
IL-6	1.29 (1.13, 1.47)	<0.001*	1.08 (0.95, 1.23)	0.27	1.31 (1.13, 1.52)	<0.001*
TNF-α	1.14 (1.01, 1.3)	0.04	0.96 (0.85, 1.09)	0.53	1.07 (0.92, 1.24)	0.4
TGF-β	1.05 (0.93, 1.17)	0.45	1.21 (1.09, 1.35)	<0.001*	1.01 (0.88, 1.15)	0.93

* Significant after Bonferroni correction for multiple comparisons (corrected p-value: 0.05/8 = 0.006).

## Discussion

A number of investigators have reported an association between inflammation and increased CV mortality in CKD.[16,17] In this study, we examined the association of circulating biomarkers of inflammation with echocardiographically determined cardiac structure and function using CRIC study participants and found significant associations between several inflammatory biomarkers and LVH and systolic dysfunction after adjusting for several traditional CV risk factors as well as measures of kidney function. Of all biomarkers, hs-CRP and IL-6 were more consistently associated with abnormal cardiac geometry and contractile dysfunction. Lower serum albumin was associated with LVMI and eccentric hypertrophy. Thus, this study shows that inflammation is a potential modulator of cardiac remodeling and function in patients with CKD.

Laboratory-based studies have shown that cytokines promote cardiac remodeling by stimulating sarcomeric protein synthesis, enhancing fetal gene expression, altering extracellular matrix degradation and triggering apoptosis.[6,18,19] Although most of the circulating cytokines are secreted from activated macrophages and lymphocytes, adipocytes and skeletal muscle are also possible sources of these biomolecules.[20,21] Proinflammatory cytokines are not constitutively expressed in the myocardium, but are upregulated in response to myocardial injury and may contribute to circulating levels.[22] CRP is a predictor of CVD in the general population and in patients with CKD.[23,24] In a cohort of resistant hypertensive patients. microalbuminuria and high CRP were independently associated with the occurrence of LVH.[25] In a small study, involving 104 maintenance hemodialysis patients, hs-CRP and systolic BP were independent predictors of LVH. [26] Whether CRP is just a marker of overall inflammatory state or a direct mediator of LVH is currently uncertain.

Based on LVMI and RWT, four patterns of cardiac geometry were recognized. Abnormal cardiac geometry is associated with CV events in patients with CKD.[27,28] In the current study, the presence of both concentric and eccentric hypertrophy was associated with elevated levels of hs-CRP and inflammatory cytokines. Circulating IL-6 was associated with the presence of both concentric and eccentric hypertrophy. In two hypertensive rat models, Kurdi et al. showed that IL-6 and leukemia inhibitory factor contributed to angiotensin II-dependent LVH. [29] *In vitro* studies show that IL-6 mediates cardiac myocyte hypertrophy by an autocrine pathway and fibroblast proliferation by a paracrine pathway.[5,30] In the current study, low serum albumin was associated with LVMI as well as with the presence of eccentric hypertrophy. A strong association between serum albumin and LV dilation has been reported in end-stage renal disease patients.[31] The link between serum albumin and cardiac geometry could be a reflection of underlying inflammation as well as other associated comorbidities such as protein energy wasting.

Heart failure may be due to systolic or diastolic dysfunction, or both.[32] In the present study, ejection fraction was negatively associated with hs-CRP and IL-6. The contractile function of isolated cardiac myocytes is modulated by cytokines through activation of the neutral sphingomyelinase pathway and by NO-mediated blunting of β-adrenergic signaling.[33,34] Pro-inflammatory cytokines may also promote diastolic heart failure through down-regulation of diastolic calcium reuptake by sarcoplasmic reticulum.[35] However, in our study only hs-CRP was associated with an increased risk for diastolic dysfunction.

The cross-sectional associations reported in this study should be interpreted with caution. Cytokines are pleiotropic in their actions, and exhibit interactive cascades in which they induce or repress their own synthesis as well as that of other cytokines and cytokine receptors.[36] An important component of the inflammatory cascade is the acute-phase response, which is regulated by cytokines such as IL-6. Zoccali et al.[37] showed that an inflammation score based on CRP, IL-6, IL-1β, IL-18 and TNF-α was not superior to IL-6 in predicting mortality in patients with ESRD. In the present study as well, IL-6 emerged as a strong and independent predictor of unfavorable cardiac geometry.

A number of studies have demonstrated that single measures of various inflammatory biomarkers at baseline are important determinants of subsequent adverse outcomes in subjects with kidney disease.[37,38] In a study involving 62 subjects without kidney disease, single measures of hs-CRP, TNF-α, IL-8, and soluble TNF receptor I and II accurately reflected the inflammatory status over a 4–6-month period.[39] However, intra-individual variation in inflammatory biomarkers is also reported in subjects with and without kidney disease.[40–42] In the Mapping of Inflammatory Markers in Chronic Kidney Disease (MIMICK) Study, inflammatory markers were measured over 3 months in 228 hemodialysis patients. Baseline CRP level was highly correlated with time-averaged CRP as well as with the median of serial CRP values.[43] However, in the multivariate Cox model, median CRP level was associated more strongly with mortality than a single baseline value, indicating that serial CRP values over time is superior in estimation of the patient's risk profile.[43]

Our study has a number of strengths which include: (a) a large cohort of patients from different races/ethnicities with a broad range of kidney function; (b) examination of a large panel of biomarkers with pro- and anti-inflammatory properties; and (c) consideration of traditional CV risk factors. Echocardiography performed using a standardized protocol, which included quality control of the measurements, is an added strength. However, these findings should be considered within the context of some limitations: (a) this is a cross-sectional analysis and hence temporal associations and causality cannot be inferred; (b) biomarkers were measured at one time point only; and (c) some echocardiographic parameters were not available in a subset of study participants due to technical difficulties.

## Conclusions

To summarize, using a large cohort of well characterized CKD subjects, this study demonstrates that abnormal cardiac structure and function are associated with specific biomarkers of inflammation. Specifically, elevated hs-CRP and IL-6 were independently and consistently associated with LVH and systolic dysfunction. Low serum albumin was associated with higher LVMI and eccentric hypertrophy. Among the pro-inflammatory biomarkers studied, IL-6 appears to best capture the inflammatory status as well as the association with adverse cardiac remodeling in CKD patients. The prognostic implications and the utility of IL-6 as a therapeutic target warrant further investigation.
